# Supplementary material for: Chemical Conversion of Hardly Ionizable Rhenium Aryl Chlorocomplexes with p-Substituted Anilines
Source: Molecules. 2021 Jun 5;26(11):3427. doi: 10.3390/molecules26113427 (PMC8201000; doi:10.3390/molecules26113427)
Supplement: Supplementary file 1 [file molecules-26-03427-s001.zip › molecules-1223012-supplementary.pdf]

Supplementary Materials

Chemical Conversion of Hardly Ionizable Rhenium Aryl Chlorocomplexes with *p*-Substituted Anilines

Martin Štícha,[1*] Ivan Jelínek,[2] Mikuláš Vlk [2]

[1] *Department of Chemistry, Faculty of Science, Charles University, Prague 2, Czech Republic*

[2] *Department of Analytical Chemistry, Faculty of Science, Charles University, Prague 2, Czech Republic*

* sticha@natur.cuni.cz

Table of contents

Figure S1. HR ESI– MS/MS spektra of complex 1 ; collision energy, CE, was 40 eV.

Figure S2. HR ESI– MS/MS spektra of complex 2 ; collision energy, CE, was 40 eV.

Figure S3. HR ESI– MS/MS spektra of complex 3 ; collision energy, CE, was 40 eV.

Figure S4. HR ESI– MS/MS spektra of complex 4 ; collision energy, CE, was 40 eV.

Figure S5. HR ESI– MS/MS spektra of complex 5 ; collision energy, CE, was 40 eV.

Figure S6. Proposed fragmentation scheme of complex 1.

Figure S7. Proposed fragmentation scheme of complex 3.

Figure S8. Proposed fragmentation scheme of complex 4.

Figure S9. Proposed fragmentation scheme of complex 5.

Figure S10. CID diagram of complex 1.

Figure S11. CID diagram of complex 3.

Figure S12. CID diagram of complex 4.

Figure S13. Graph of calculated bond elongation of prepared complexes.

Table S1. Cartesian coordinates calculated for the optimized neutral structure of complex 1.

Table S2.Cartesian coordinates calculated for the optimized neutral structure of complex 2.

Table S3.Cartesian coordinates calculated for the optimized neutral structure of complex 3.

Table S4.Cartesian coordinates calculated for the optimized neutral structure of complex 4.

Table S5. Cartesian coordinates calculated for the optimized neutral structure of complex 5.

Table S6. Chemical names, labels, and formulas of prepared rhenium complexes.

Table S7. Calculated bond elongation of prepared complexes.

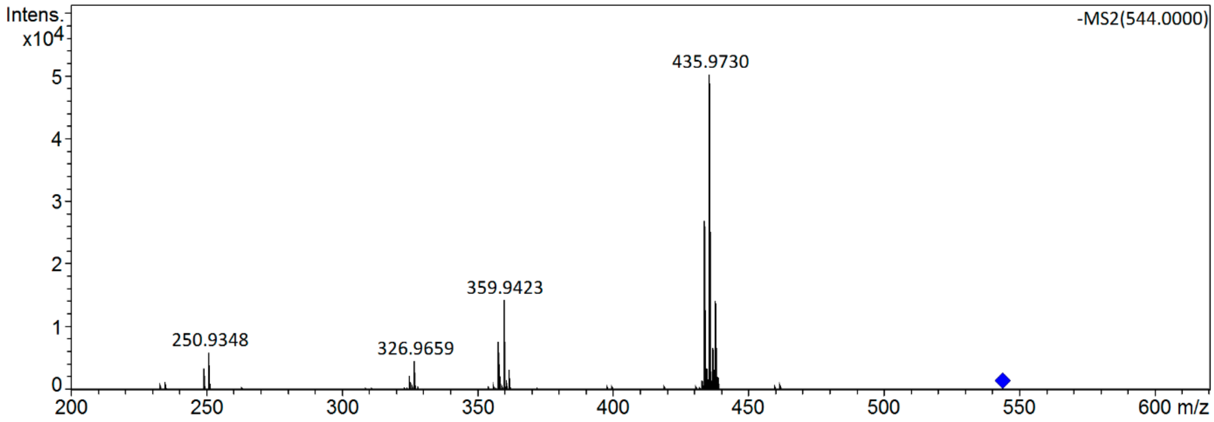

Figure S1. HR ESI– MS/MS spektra of complex 1; collision energy, CE, was 40 eV.

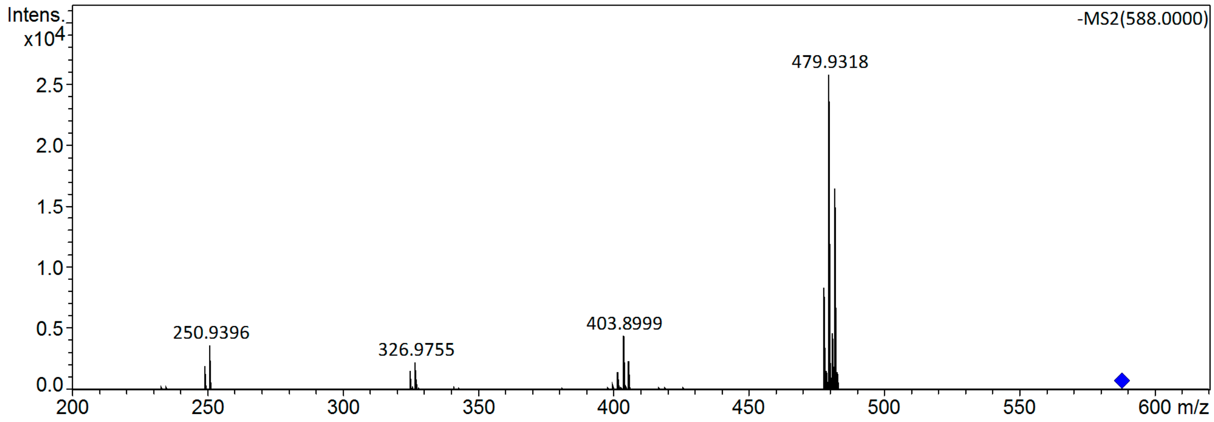

Figure S2. HR ESI– MS/MS spektra of complex 2; collision energy, CE, was 40 eV.

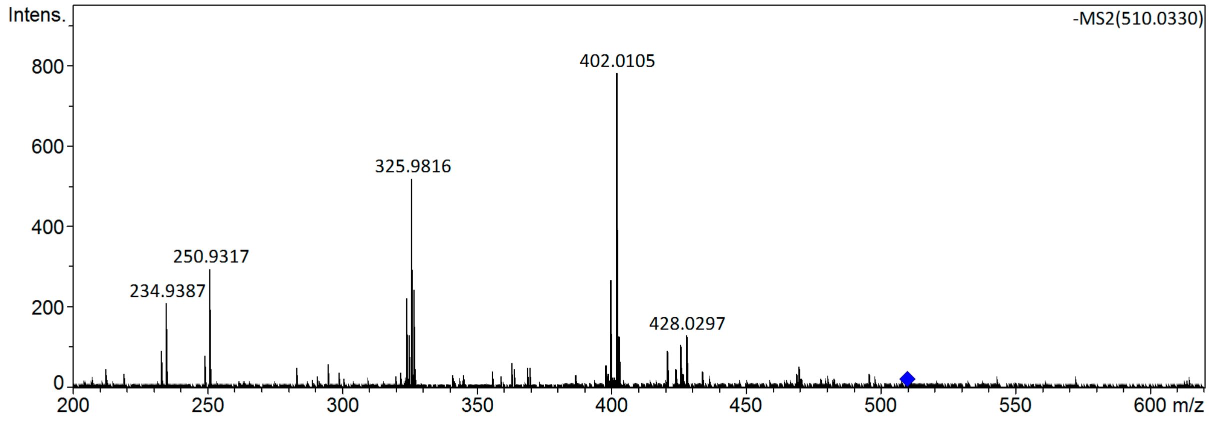

Figure S3. HR ESI– MS/MS spektra of complex 3; collision energy, CE, was 40 eV.

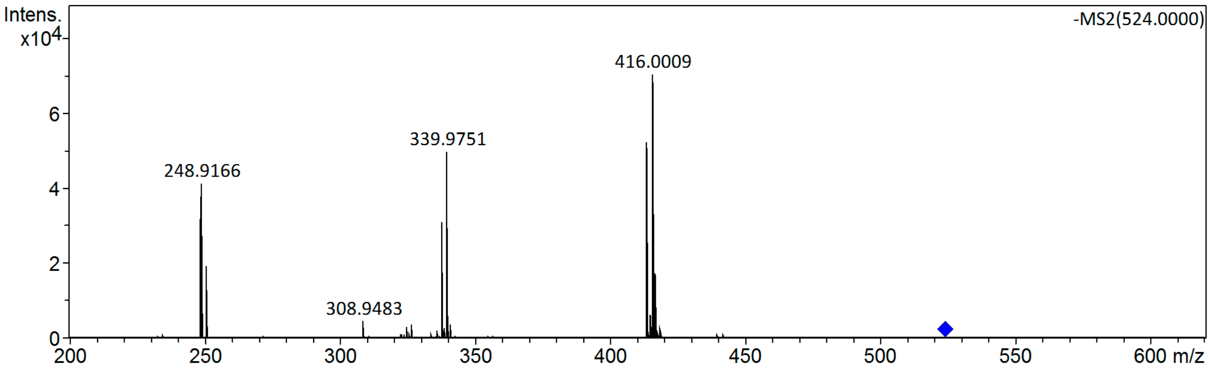

Figure S4. HR ESI– MS/MS spektra of complex 4; collision energy, CE, was 40 eV.

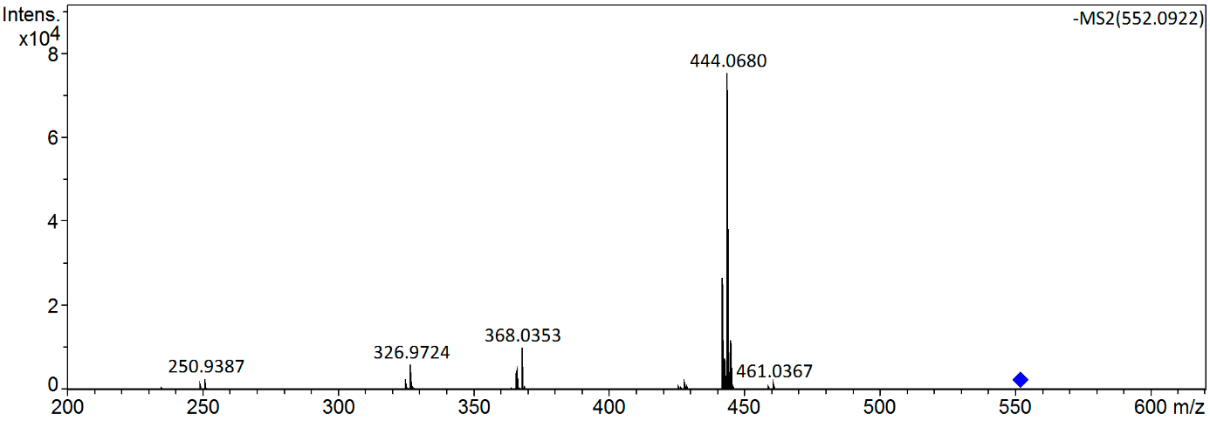

Figure S5. HR ESI– MS/MS spektra of complex 5; collision energy, CE, was 40 eV.

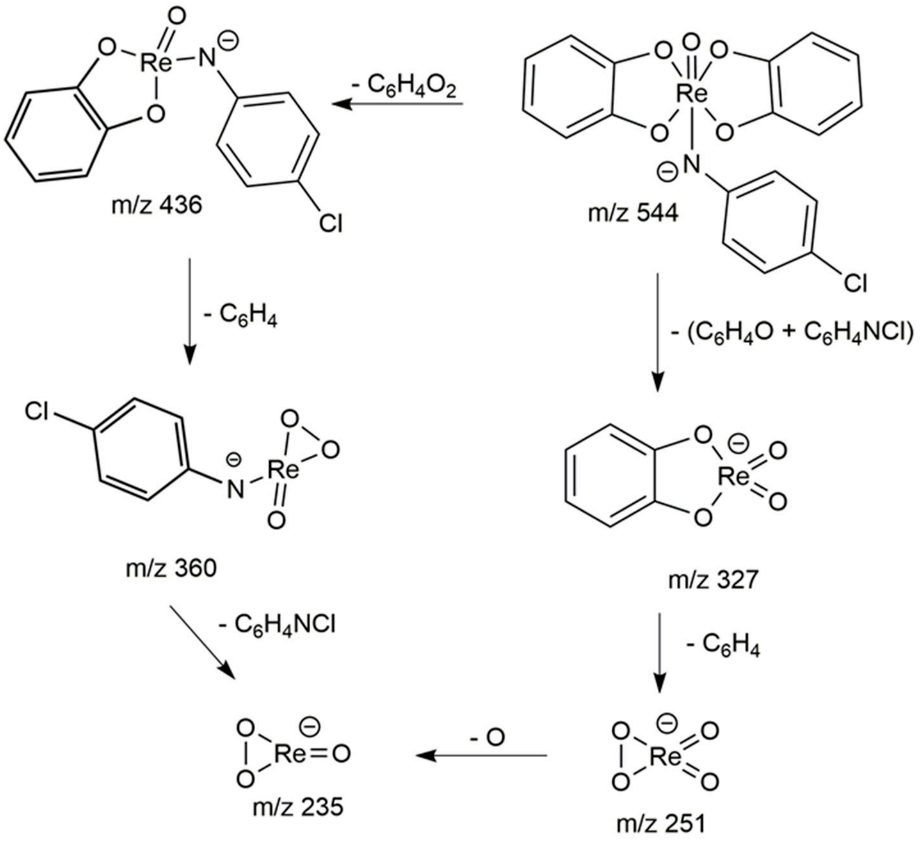

Figure S6. Proposed fragmentation scheme of complex 1.

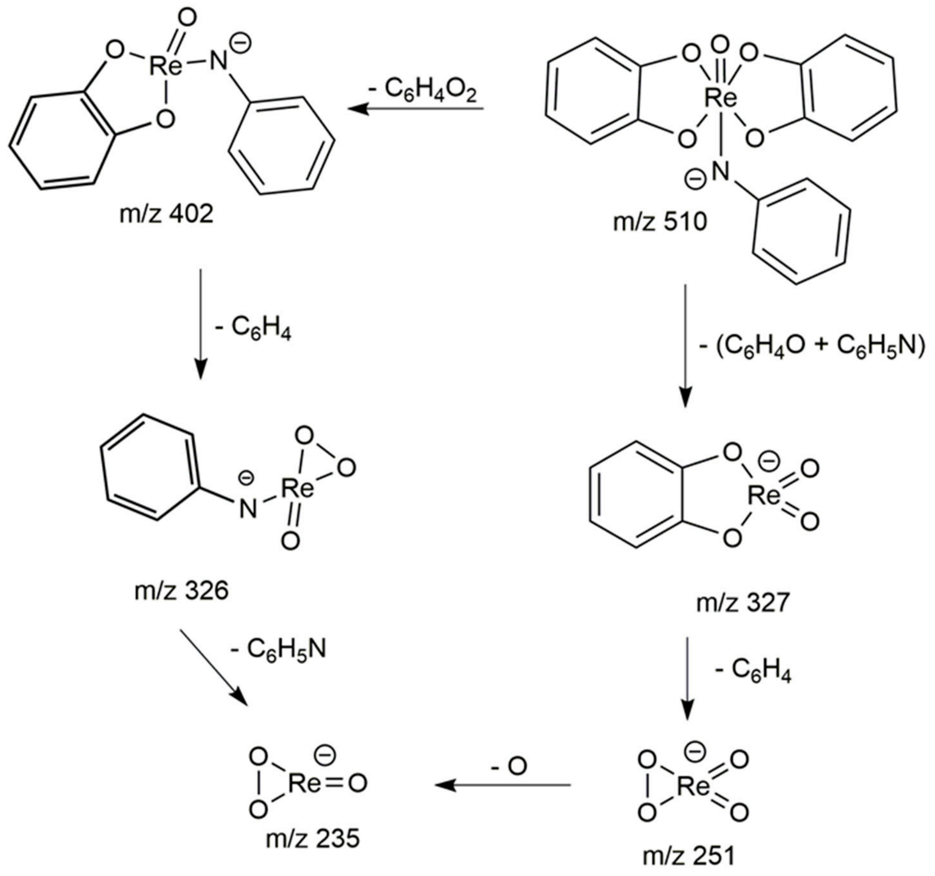

Figure S7. Proposed fragmentation scheme of complex 3

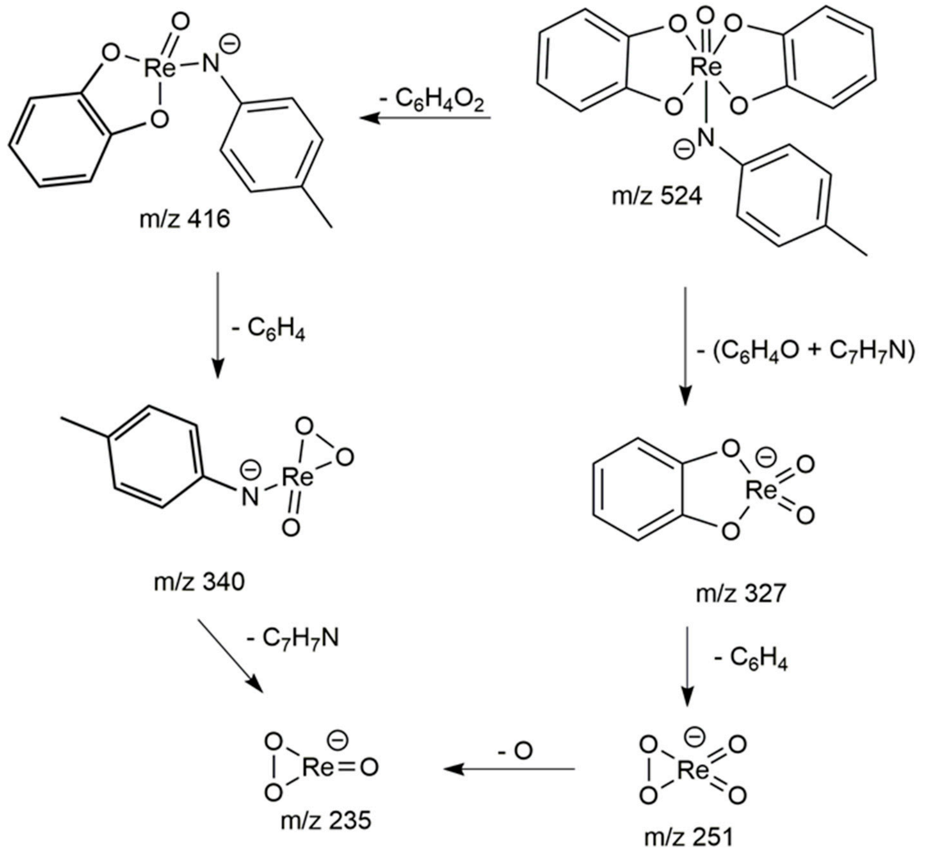

Figure S8. Proposed fragmentation scheme of complex 4

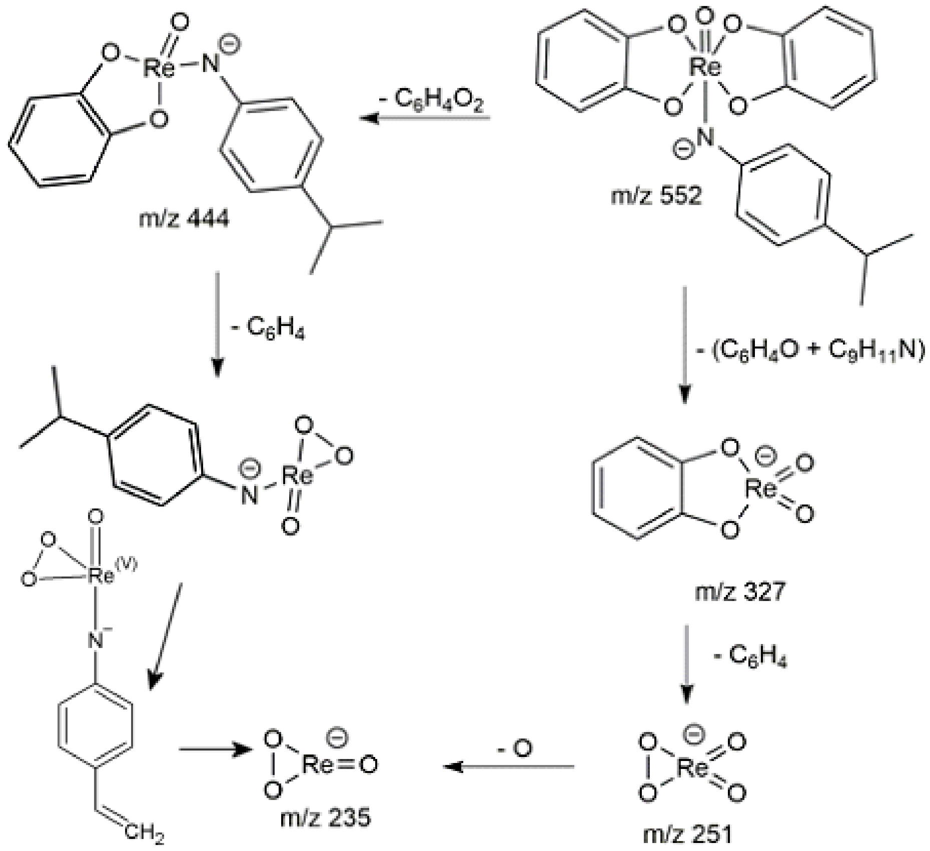

Figure S9. Proposed fragmentation scheme of complex 5

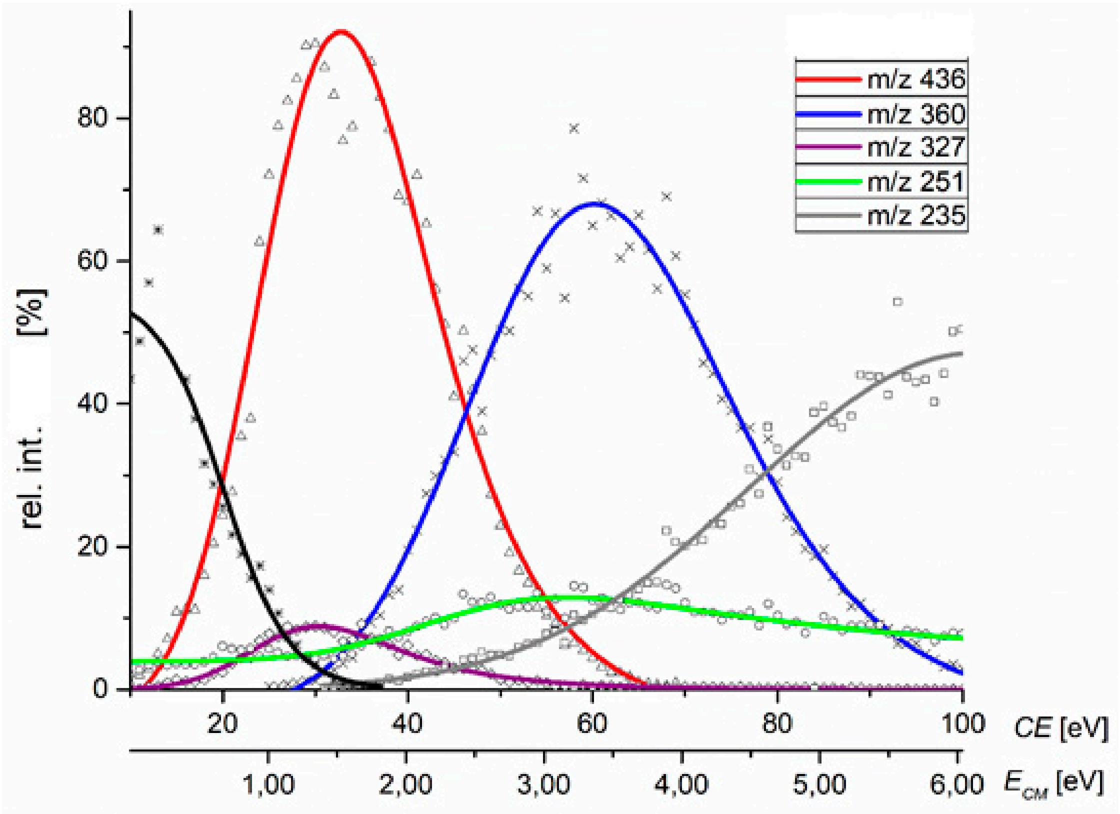

Figure S10. CID diagram of complex 1

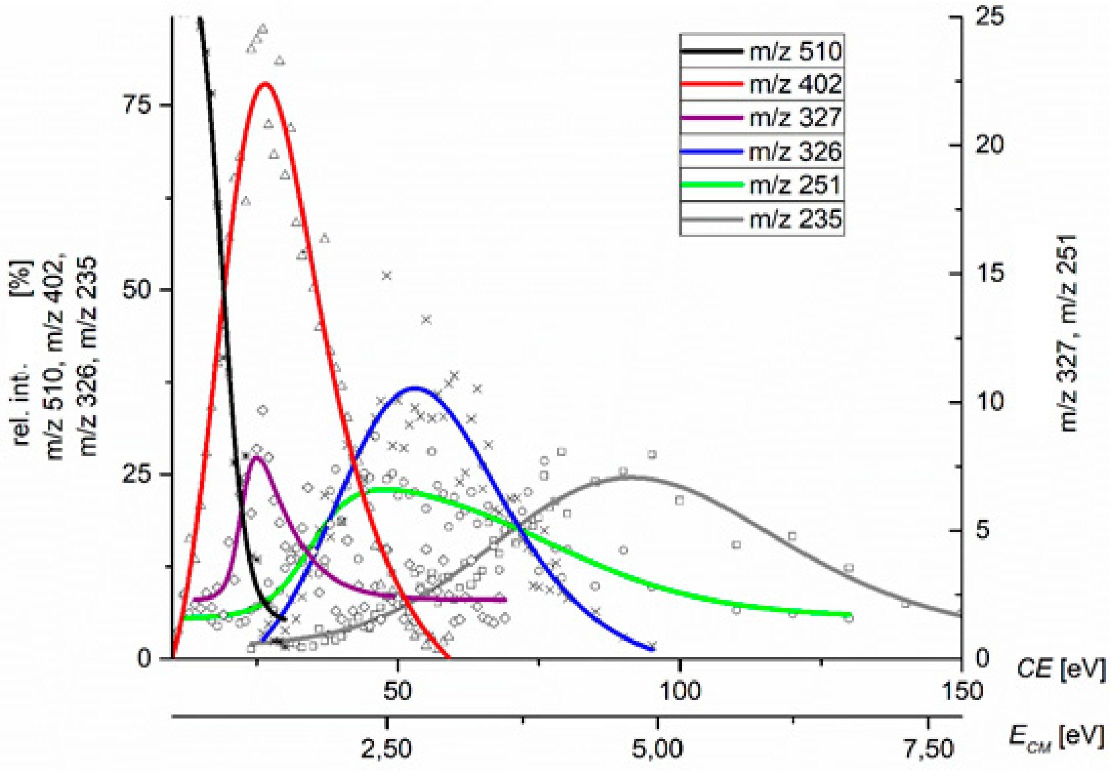

Figure S11. CID diagram of complex 3

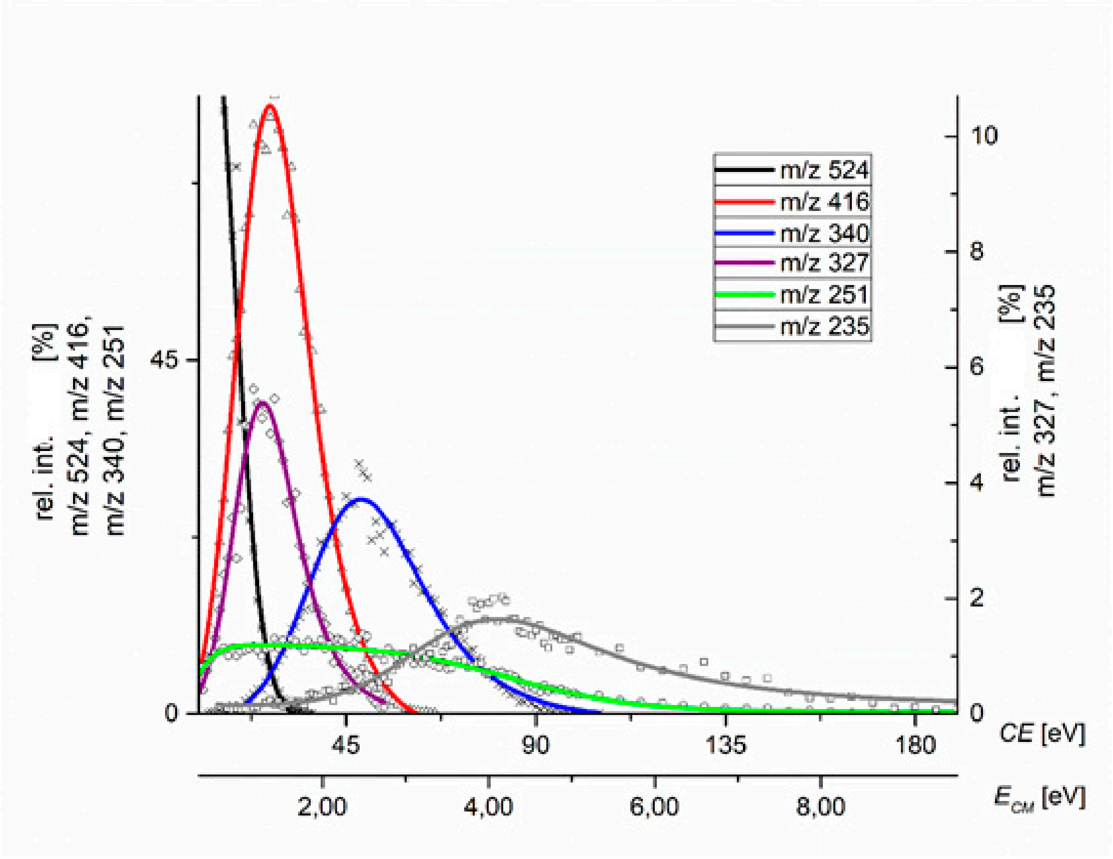

Figure S12. CID diagram of complex 4

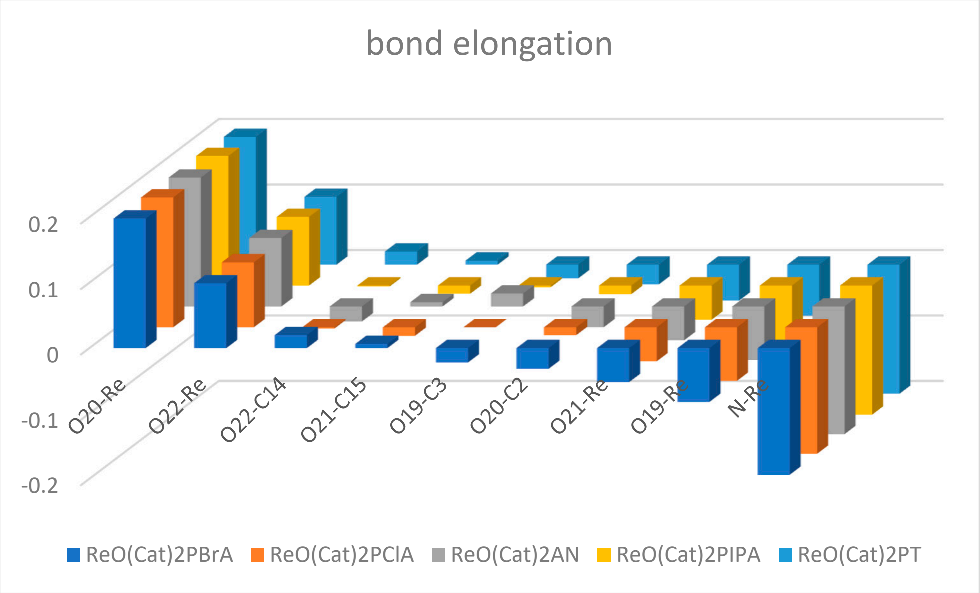

Figure S13. Graph of calculated bond elongation of prepared complexes.

Table S1. Cartesian coordinates calculated for the optimized neutral structure of complex 1.

Input orientation:

Center Number	Atomic Number	Atomic Type	Coordinates (Angstroms) X	Y	Z
1	6	0	5.542844	0.634768	0.330638
2	6	0	5.333374	-0.228210	1.433435
3	6	0	4.068715	-0.802056	1.671272
4	6	0	3.042556	-0.483579	0.776820
5	6	0	3.243373	0.370372	-0.333618
6	6	0	4.501732	0.950356	-0.563964
7	6	0	-2.461768	-1.075022	-0.484426
8	6	0	-1.908257	-1.884622	0.538139
9	6	0	-2.663186	-2.839711	1.229027
10	6	0	-4.018191	-2.965728	0.871484
11	6	0	-4.585946	-2.146348	-0.138025
12	6	0	-3.822829	-1.180954	-0.818852
13	8	0	2.121291	0.538634	-1.094926
14	8	0	1.735225	-0.933728	0.860682
15	8	0	-1.558712	-0.209201	-1.019813
16	8	0	-0.587901	-1.559890	0.789217
17	75	0	0.395234	-0.416098	-0.446351
18	8	0	0.591228	-1.358134	-1.898465
19	6	0	-1.989149	1.820752	1.119712
20	6	0	-3.080841	2.623249	1.478496
21	6	0	-3.268120	3.886376	0.879111
22	6	0	-2.345833	4.346562	-0.083010
23	6	0	-1.250757	3.549753	-0.448826

24	6	0	-1.063389	2.275499	0.147368
25	7	0	0.080100	1.520576	-0.179237
26	1	0	6.529739	1.059717	0.165941
27	1	0	6.157328	-0.451868	2.105412
28	1	0	3.882026	-1.464161	2.510523
29	1	0	4.657131	1.602218	-1.417691
30	1	0	-2.208609	-3.447442	2.004576
31	1	0	-4.638699	-3.700286	1.377320
32	1	0	-5.634495	-2.271092	-0.395977
33	1	0	-4.247332	-0.550414	-1.592927
34	1	0	-1.829047	0.864330	1.608095
35	1	0	-3.781765	2.269159	2.229936
36	1	0	-2.482727	5.319903	-0.547289
37	1	0	-0.545739	3.898279	-1.201026
38	17	0	-4.641158	4.887768	1.336911
39	1	0	0.619836	1.765067	0.954570

Table S2. Cartesian coordinates calculated for the optimized neutral structure of complex 2.

Center Number	Atomic Number	Atomic Type	Coordinates (Angstroms) X	Y	Z
1	6	0	-4.063120	-0.822081	1.673164
2	6	0	-3.037877	-0.499696	0.779060
3	6	0	-3.240724	0.356227	-0.329489
4	6	0	-4.500288	0.934244	-0.558189
5	6	0	-5.540488	0.614719	0.336074
6	6	0	-5.328973	-0.250198	1.436961
7	1	0	-3.874864	-1.485608	2.510940
8	1	0	-6.528264	1.038091	0.172604
9	1	0	-6.152269	-0.476894	2.108726
10	6	0	2.672592	-2.845660	1.224387
11	6	0	4.027724	-2.968270	0.866138
12	6	0	4.593551	-2.145630	-0.141799
13	6	0	3.828331	-1.180272	-0.820315
14	6	0	2.467176	-1.077710	-0.485224
15	6	0	1.915578	-1.890572	0.535785
16	1	0	4.649828	-3.702695	1.370204
17	1	0	5.642256	-2.267776	-0.400355
18	1	0	4.251352	-0.547253	-1.593178
19	8	0	-2.119222	0.528300	-1.090799
20	8	0	-1.729643	-0.947471	0.861538
21	8	0	0.594673	-1.568952	0.787982
22	8	0	1.562258	-0.212510	-1.018468
23	8	0	-0.585724	-1.363758	-1.898880
24	75	0	-0.391093	-0.424443	-0.444822

25	1	0	-4.657237	1.587620	-1.410474
26	1	0	2.219455	-3.455932	1.998783
27	6	0	1.062471	2.268726	0.154158
28	6	0	1.247158	3.544608	-0.439383
29	6	0	2.340798	4.342772	-0.072227
30	6	0	3.264297	3.882339	0.888614
31	6	0	3.079678	2.617577	1.485368
32	6	0	1.989435	1.813717	1.125231
33	1	0	0.541215	3.893358	-1.190610
34	1	0	2.475643	5.317364	-0.534477
35	1	0	3.781538	2.263256	2.235826
36	1	0	1.831359	0.855947	1.611629
37	7	0	-0.079651	1.512270	-0.173683
38	1	0	-0.844224	2.072103	-0.555460
39	35	0	4.752397	4.970922	1.387259

Table S3. Cartesian coordinates calculated for the optimized neutral structure of complex 3.

Center Number	Atomic Number	Atomic Type	Coordinates (Angstroms) X	Y	Z
1	6	0	-5.503665	0.619118	-0.417995
2	6	0	-5.272530	-0.298844	-1.444051
3	6	0	-3.997197	-0.850686	-1.657932
4	6	0	-2.976104	-0.446975	-0.808412
5	6	0	-3.208607	0.490725	0.246751
6	6	0	-4.475102	1.032016	0.445606
7	6	0	2.338411	-1.066907	0.554059
8	6	0	1.760862	-1.818623	-0.515814
9	6	0	2.493059	-2.770799	-1.213134
10	6	0	3.830048	-2.962088	-0.826945
11	6	0	4.401333	-2.222613	0.212784
12	6	0	3.667625	-1.257821	0.920882
13	8	0	-2.124800	0.781686	0.975455
14	8	0	-1.695355	-0.868402	-0.899448
15	8	0	1.490780	-0.193647	1.111506
16	8	0	0.478405	-1.479462	-0.776911
17	75	0	-0.438529	-0.297584	0.513150
18	8	0	-0.769616	-1.167986	1.923151
19	6	0	1.899900	1.555041	-1.300597
20	6	0	3.115252	2.107022	-1.711273
21	6	0	3.641995	3.231804	-1.068705
22	6	0	2.947427	3.811388	-0.001369
23	6	0	1.735144	3.272554	0.429766
24	6	0	1.210461	2.133798	-0.216401

25	7	0	-0.049292	1.595891	0.190364
26	1	0	-6.503627	1.027578	-0.275784
27	1	0	-6.091643	-0.599636	-2.095002
28	1	0	-3.808137	-1.566384	-2.453663
29	1	0	-4.653843	1.740756	1.248666
30	1	0	2.040587	-3.339781	-2.021147
31	1	0	4.428820	-3.704614	-1.352055
32	1	0	5.441691	-2.396537	0.486735
33	1	0	4.107113	-0.679774	1.729108
34	1	0	1.496985	0.690452	-1.829894
35	1	0	3.654636	1.655570	-2.544246
36	1	0	4.588421	3.654564	-1.397319
37	1	0	3.356465	4.687976	0.500511
38	1	0	1.207488	3.719859	1.268892
39	1	0	-0.715466	2.331344	0.483086

Table S4. Cartesian coordinates calculated for the optimized neutral structure of complex 4.

Center Number	Atomic Number	Atomic Type	Coordinates (Angstroms) X	Y	Z
1	6	0	4.594446	-1.223763	1.885324
2	6	0	3.630113	-0.921844	0.919151
3	6	0	3.489533	-1.672997	-0.271846
4	6	0	4.319007	-2.779940	-0.515151
5	6	0	5.295221	-3.091341	0.451031
6	6	0	5.433892	-2.327128	1.635131
7	1	0	4.678620	-0.624627	2.786118
8	1	0	5.959985	-3.934111	0.279652
9	1	0	6.197178	-2.593954	2.360776
10	6	0	-0.190822	3.920480	1.390666
11	6	0	-1.257372	4.742570	0.983292
12	6	0	-2.044858	4.409672	-0.149147
13	6	0	-1.793482	3.251991	-0.906876
14	6	0	-0.708778	2.446186	-0.519939
15	6	0	0.059833	2.776485	0.623803
16	1	0	-1.482321	5.644543	1.545588
17	1	0	-2.858862	5.067754	-0.441889
18	1	0	-2.389025	2.990015	-1.774996
19	8	0	2.500038	-1.204566	-1.089878
20	8	0	2.703890	0.104927	1.000890
21	8	0	1.007771	1.808180	0.899741
22	8	0	-0.303024	1.290037	-1.112612
23	8	0	2.210597	1.267547	-1.730836
24	75	0	1.422590	0.434686	-0.419687

25	1	0	4.213630	-3.354396	-1.429866
26	1	0	0.418832	4.148234	2.258897
27	6	0	-1.237000	-1.184782	-0.247753
28	6	0	-1.970365	-2.131927	-1.007792
29	6	0	-3.350573	-2.290487	-0.815126
30	6	0	-4.004846	-1.496724	0.143386
31	6	0	-3.296985	-0.556464	0.914067
32	6	0	-1.918212	-0.402748	0.718128
33	1	0	-1.460772	-2.732557	-1.757943
34	1	0	-3.903990	-3.014793	-1.404362
35	1	0	-3.808929	0.041698	1.661021
36	1	0	-1.364080	0.297628	1.334869
37	7	0	0.157412	-1.092274	-0.409949
38	1	0	0.579656	-1.930680	-0.812784
39	6	0	-5.520446	-1.664908	0.358496
40	1	0	-6.049234	-1.183892	-0.437686
41	1	0	-5.765459	-2.706399	0.371333
42	1	0	-5.799689	-1.221288	1.291300

Table S5. Cartesian coordinates calculated for the optimized neutral structure of complex 5.

Center	Atomic	Atomic	Coordinates (Angstroms)		
Number	Number	Type	X	Y	Z
1	6	0	-5.274891	-2.854316	0.507536
2	6	0	-5.383488	-2.043586	1.663255
3	6	0	-4.490584	-0.975945	1.882863
4	6	0	-3.504375	-0.756903	0.916527
5	6	0	-3.392647	-1.555305	-0.246554
6	6	0	-4.276615	-2.625928	-0.459497
7	6	0	1.045716	2.299609	-0.564375
8	6	0	0.273443	2.724611	0.544894
9	6	0	0.578110	3.881971	1.270800
10	6	0	1.703083	4.619266	0.857807
11	6	0	2.493522	4.191300	-0.239914
12	6	0	2.186851	3.020511	-0.956194
13	8	0	-2.376898	-1.165626	-1.073211
14	8	0	-2.530688	0.226937	0.970894
15	8	0	0.581594	1.147917	-1.121169
16	8	0	-0.740871	1.830637	0.834462
17	75	0	-1.208700	0.428836	-0.437375
18	8	0	-1.917159	1.252253	-1.799292
19	6	0	2.036768	-0.574681	0.827798
20	6	0	3.395963	-0.818981	1.067922
21	6	0	4.076234	-1.833377	0.362270
22	6	0	3.381000	-2.611025	-0.586394
23	6	0	2.020841	-2.372938	-0.833787
24	6	0	1.335235	-1.348439	-0.130408
25	7	0	-0.046401	-1.171431	-0.339694
26	1	0	-5.980157	-3.668079	0.358743

27	1	0	-6.164835	-2.245867	2.390563
28	1	0	-4.551302	-0.341977	2.761379
29	1	0	-4.193656	-3.236068	-1.353260
30	1	0	-0.035813	4.183113	2.113340
31	1	0	1.970972	5.528550	1.388756
32	1	0	3.353487	4.785232	-0.538716
33	1	0	2.784167	2.685510	-1.797609
34	1	0	1.508107	0.183630	1.397370
35	1	0	3.923980	-0.225164	1.809361
36	1	0	3.898388	-3.395971	-1.132202
37	1	0	1.486565	-2.963701	-1.575371
38	6	0	5.773882	-2.091804	0.476317
39	6	0	6.330231	-3.239527	-0.525877
40	1	0	5.879747	-4.178003	-0.278587
41	1	0	7.392697	-3.316355	-0.425367
42	1	0	6.085211	-2.981196	-1.534947
43	6	0	6.752344	-1.196838	1.562949
44	1	0	6.260473	-1.107345	-0.650792
45	1	0	6.141948	-0.506280	2.106527
46	1	0	7.496276	-0.659147	1.013069
47	1	0	7.229475	-1.866561	2.247606
48	1	0	-0.542404	-2.030712	-0.464664

Table S6. Chemical names, labels, and formulas of prepared rhenium complexes.

Entry	Name	Label	Formula
1	N-(bis(1,2-dihydroxybenzene)oxorhenium)-parachloroaniline	$[Re^{VII}(O)(Cat)_2PClA]^{-a}$	$C_{18}H_{12}ClNO_5Re$
2	N-(bis(1,2-dihydroxybenzene)oxorhenium)-parabromoaniline	$[Re^{VII}(O)(Cat)_2PBrA]^{-a}$	$C_{18}H_{12}BrNO_5Re$
3	N-(bis(1,2-dihydroxybenzene)oxorhenium)-aniline	$[Re^{VII}(O)(Cat)_2An]^{-a}$	$C_{18}H_{13}NO_5Re$
4	N-(bis(1,2-dihydroxybenzene)oxorhenium)-paratoluidine	$[Re^{VII}(O)(Cat)_2PT]^{-a}$	$C_{19}H_{15}NO_5Re$
5	N-(bis(1,2-dihydroxybenzene)oxorhenium)-paracumidine	$[Re^{VII}(O)(Cat)_2PIPA]^{-a}$	$C_{21}H_{19}NO_5Re$

Table S7. Calculated bond elongation of prepared complexes.

	ReO(Cat)2PBrA	ReO(Cat)2PClA	ReO(Cat)2AN	ReO(Cat)2PIPA	ReO(Cat)2PT
**O20-Re**	0.1979	0.1983	0.1967	0.1978	0.19499
**O22-Re**	0.0992	0.0992	0.1043	0.1048	0.10348
**O22-C14**	0.0199	-0.0019	-0.0229	-0.0014	0.02013
**O21-C15**	0.0064	-0.013	0.0066	-0.0125	0.00623
**O19-C3**	-0.022	-0.0002	0.0197	-0.0025	-0.02089
**O20-C2**	-0.0317	-0.0121	-0.0318	-0.0131	-0.03019
**O21-Re**	-0.052	-0.0521	-0.0518	-0.0522	-0.05489
**O19-Re**	-0.0823	-0.082	-0.082	-0.0836	-0.07794
**N-Re**	-0.1938	-0.1935	-0.1953	-0.1975	-0.19741
